# Predictors of COVID-19-related health anxiety among health care workers: a cross-sectional study

**DOI:** 10.1186/s40359-022-00880-y

**Published:** 2022-07-11

**Authors:** Maryam Saeedi, Sahar Yazdi, Rasoul Corani Bahador

**Affiliations:** Saveh University of Medical Sciences, Saveh, Iran

**Keywords:** Anxiety, Health Personnel, Coronavirus, COVID-19

## Abstract

**Background:**

One of the psychological issues that may affect health care workers (HCWs) during the outbreak of COVID-19 is health anxiety. Health anxiety disorder goes beyond normal health concerns and can seriously affect occupational and interpersonal performance. The present study was designed to determine the level of COVID-19-related health anxiety and its predictors in Iranian HCWs.

**Methods:**

This is a cross-sectional study. Data were collected online through a demographic information questionnaire and the short version of the Health Anxiety Questionnaire. The online questionnaires were created via Google Form and the URL link was sent to HCWs via email or social networking applications. In total, questionnaires were sent to more than 1,500 HCWs throughout Iran. Data were analyzed with SPSS software version 23.

**Results:**

Five hundred and fifty-two HCWs completed and sent the questionnaires. The mean scores of health anxiety of HCWs were higher than the cutoff point of health anxiety (17.28 ± 8.84) and 58.1% of HCWs had health anxiety. There was a significant inverse relationship between health anxiety score and age (r = − 0.13; *P* = 0.002), work experience (r = − 0.16; *P* < 0.001) and income level (r = − 0.097; *P* = 0.03). The rate of health anxiety was significantly higher in females (*P* = 0.03). Based on regression results, age and hospital category were significant risk factors for health anxiety.

**Conclusion:**

Based on the results of this study, employees working in health centers in Iran had high health anxiety. Due to the high level of health anxiety in HCWs, it is important to consider strategies to reduce their health anxiety in the current situation.

## Background

The COVID-19 outbreak has exposed health care workers (HCWs) to high stress due to the high risk of infection, isolation, caring for critically ill patients, and overwork. This difficult situation leads to mental health problems such as anxiety, fear, stress, insomnia, and depression [5–7]. One of the psychological issues that may affect HCWs during the outbreak of COVID-19 is health anxiety. Health anxiety is a disorder characterized by a great deal of fear and anxiety about having a serious illness [8]. Health anxiety is a continuous concept that on the one hand is a constant health concern, and on the other hand is hypochondriasis, which is characterized by extreme fears about health and sometimes physical symptoms [9]. Anyone can experience health anxiety during their lives, and in some cases, it can be persistent and distressing [10].

The American Psychological Association lists health anxiety as a relatively common disorder that persists chronically if left untreated, leading to overuse of the health care system and dysfunction [11, 12]. Health anxiety disorder is a serious problem that can affect the quality of life and impose considerable costs on public health [13]. The health anxiety disorder goes beyond normal health concerns and can seriously affect occupational and interpersonal performance [14].

Health anxiety is a relatively new diagnosis, so its epidemiology is unclear, but it appears to be present in about 1 in 16 populations at some point in their lives. Health anxiety is often found in combination with generalized anxiety, obsessive–compulsive disorder, and a wide range of somatic disorders and is classified differently [15]. In Iranian society, 43.8% of people had mild to moderate and 19.1% had severe COVID-19-related anxiety [16]. The prevalence of health anxiety in the general population was lower than the medical staff [17]. The prevalence of health anxiety is higher in medical employees [18]. In recent studies, the prevalence of health anxiety in medical staff has been reported 30.14 to 72.3% [18–22].

Based on the health belief model, personal perceptions and beliefs affect susceptibility to disease [23]. Stress and anxiety can weaken the immune system and make a person vulnerable to disease [24]. Health anxiety along with high workload can lead to burnout and psychological distress in HCWs [25, 26]. Healthcare workers are at the forefront of the fight against COVID-19 and play a key role in controlling the epidemic. Psychological distress can expose healthcare workers to mental disorders and affect the quality of health care delivery [27]. Therefore, maintaining their health is not only important from an individual point of view but also ensures the health of the general public [28].

A review of the literature reveals that there is no agreement on the predictors of health anxiety in HCWs. Studies have reported various results on the relationship between demographic variables and health anxiety in HCWs. For example, in some studies, age and educational level have been determined as predictors of health anxiety [29, 30], while in other studies, a significant relationship between these factors and health anxiety has not been reported [20, 21, 31]. In most studies, the level of health anxiety was higher in women [19, 32, 33]. While in the study of Mirzabeigi et al. [20], the rate of anxiety was higher in men. In some studies, no significant relationship was found between gender and health anxiety [21, 31]. In most studies, health anxiety was higher in nurses [20, 22, 30, 34], but in some studies, this finding was not obtained [19, 32]. In some studies, factors such as working on the frontline [31, 35], and having an underlying disease [19, 21] have been reported as predictors of health anxiety in health workers. But in other studies, such results were not obtained [30, 31].

Previous studies have examined the relationship between a limited number of demographic variables and COVID-19-related health anxiety of HCW. While in the present study, we performed a comprehensive study on the effect of demographic variables such as age, gender, marital status, having children, having underlying disease, level of education, hospital category, career type, level of income, and work history on health anxiety of HCW.

Studies have been performed in Iran to investigate the health anxiety of HCWs, but these studies have mostly been performed on a limited number of HCWs or centers [20, 21, 36]. Therefore, it is necessary to conduct a study of a larger number of health care workers and various centers. As the number of COVID-19 cases continues to rise worldwide, and health anxiety is one of the psychological disorders affecting HCWs in the current situation; more research on health anxiety and its related factors is needed to plan for appropriate strategies to deal with it. Accordingly, the present study was designed to determine the level of COVID-19-related health anxiety and its predictors in Iranian HCWs. Therefore, the study was developed to respond to these questions: (1) What is the prevalence of Covid-19-related health anxiety among HCWs? (2) What are the predictors of health anxiety in health care workers?

## Methods

### Study design and participants

This is a cross-sectional study conducted through an online survey from May 12 to July 12, 2020. The STROBE guidelines were applied to improve the quality of the article. The study population included all health care workers, including physicians, nurses, midwives, operating room technicians, anesthesiologists, and laboratory scientists who were employed in Iranian hospitals at the time of the outbreak of COVID-19 in Iran.

Inclusion criteria included the following: 1- Willingness to participate in the study. 2- Being employed in Iranian hospitals, including public, private, and semi-private hospitals that provided for COVID-19 patients during the outbreak of COVID-19 disease in Iran. 3- At least six months have passed from the date of their employment. 4- Be engaged in health services in the mentioned fields. The samples were excluded from the study if they did not complete the research questionnaires completely, left the service, were inactive at the time of the COVID-19 outbreak, or engaged in work other than the mentioned fields.

The online questionnaire was created via Google Form and the URL link was sent to health care staff via email or social networking applications such as Telegram or WhatsApp. After clicking on the link to the questionnaire, the participants were transferred to a page that included the purpose of the research and the consent to participate in the research. If the participant intended to participate in the research and clicked on the confirmation option, the questionnaire statements were uploaded for the participants.

The sample size was calculated using the G * Power 3.1.9.2 statistical program. The calculation was based on a small effect size F^2^ of 0.05; probability of error (alpha) of 5%; a power of 95% and ten predictors of health anxiety. Based on these parameters, the required sample size was calculated to be at least 256. Due to the availability of samples, more samples have entered to the research.

In total, questionnaires were sent to more than 1,500 health care employees throughout Iran and 552 employees completed and sent the questionnaires. The response rate was about 36.8%. The data in the Excel file was extracted from the Google form and prepared for analysis.

### Data collection tool

Data were collected online through a demographic information questionnaire and the short version of the Health Anxiety Questionnaire. The questions of the demographic information questionnaire included age, gender, marital status, having children, having underlying disease, level of education, place of work, hospital category, career type, level of income, and work history. The short version of the Health Anxiety Questionnaire was designed by Salkovskis and Warwick and consists of 18 items. Each item has four options and each option has a score between 0 and 3. The total score of the questionnaire is from 0 to 54, and higher scores indicate a higher level of anxiety [37]. Also, the cutoff point for health anxiety is 15, and a score of 15 and above means having health anxiety[19]. The questionnaire has a two-factor structure, including the probability of getting the disease (14 questions) and the negative consequences of getting the disease (4 questions). Cronbach's alpha coefficient of the questionnaire was reported between 0.7 and 0.92 by its designer[37]. This questionnaire has also been translated and validated in Persian in Iranian studies [38–40].

### Statistical analysis

Statistical analyses were performed using SPSS software version 23. Descriptive statistical methods such as determining the mean, standard deviation, and frequency were used to describe the demographic variables and scores of health anxiety. The Chi-squared test was used to compare demographic variables in samples with health anxiety and without health anxiety. The Spearman correlation test was used to examine the correlation between participants' demographic variables such as age, work experience, and income level with the total score of health anxiety. The Mann—Whitney test was used to evaluate the effect of two-state qualitative variables such as gender on the total score of health anxiety. The Kruskal—Wallis test was applied to compare health anxiety scores in terms of variables in more than two states. Logistic regression was used to investigate the predictive effect of demographic variables on health anxiety. The significance level of 0.05 (two-tailed) was determined for all analyses.

### Ethical review

This study has ethical approval from the Ethics Committee of Saveh University of Medical Sciences (Code: IR.SAVEHUMS.REC.1399.008). Informed consent was obtained from all participants in the study. All procedures were performed under relevant guidelines.

## Results

Five hundred and fifty-two HCWs completed and sent the questionnaires. The response rate was about 36.8%. The demographic information of the samples is presented in Table 1. Most of the samples were female (76.8%), married (72.6%), nurses (44.6%), had children (56.9%) and bachelor's degree (69.3%), worked in public hospitals (76.3%), had no underlying disease (87.4%), had an income level between 30 to 60 million rials (65.6%), were in the age range of 31–40 years (37.8%) and had work experience of one to 10 years (49.8%).

**Table1:** The comparison of demographic and occupational variables in participants with or without health anxiety

Variables	N (%)	HA n = 214	Non-HA n = 297	λ^2^	*P*
Gender					
Female	418 (76.8)	236	150	6.16	0.009
Male	126 (23.2)	58	62		
Age category					
20–30	177 (33.1)	104	66	12.85	0.005
31–40	202 (37.8)	119	66		
41–50	136 (25.4)	58	66		
51–60	20 (3.7)	7	11		
Marital status					
Single	149 (27.4)	83	59	0.01	0.92
Married	395 (72.6)	211	153		
Having children					
Yes	309 (56.9)	162	118	0.006	0.5
No	234 (43.1)	130	96		
Career type					
Nurse	242 (43.8)	46	37	0.35	0.83
Physician	90 (16.6)	133	95		
Other	194 (39.6)	115	79		
Level of education					
Undergraduate	60 (11)	27	28	6.21	0.045
Bachelor	377 (69.3)	216	135		
MA and above	107 (19.7)	50	50		
Having disease					
Yes	67 (12.3)	37	25	1.56	0.45
No	477 (87.4)	256	189		
Working years					
01-Oct	275 (49.8)	166	39	13.85	0.003
Nov-20	181 (33.8)	93	71		
21–30	74 (13.8)	82	42		
> 30	5	3	1		
Hospital category					
Public	411 (76.3)	210	172	6.39	0.041
Private	38 (7.1)	25	12		
Semiprivate	90 (16.7)	56	26		
Income level (million rials)					
Oct-30	146 (26.8)	91	48	7.56	0.182
31–60	357 (65.5)	182	150		
61–80	35 (6.4)	17	13		
81–100	7 (1.3)	3	2		

The mean scores of the health anxiety of HCWs were 17.28 ± 8.84 which was higher than the cutoff point of health anxiety and 58.1% of HCWs had COVID-19-related health anxiety. There was a statistically significant difference between groups with health anxiety (HA) and without health anxiety (non-HA) in terms of gender (λ^2^ = 6.16, *P* = 0.009), age groups (λ^2^ = 12.85, *P* = 0.005), Level of education (λ^2^ = 6.21, *P* = 0.045), work experience (λ^2^ = 13.85, *P* = 0.003), hospital category (λ^2^ = 6.39, *P* = 0.041) (Table 1).

There was also a significant inverse relationship between health anxiety total score and age (r = -0.13; *P* = 0.002), work experience (r = -0.16; *P* < 0.001) and income level (r = − 0.097; *P* = 0.03). The results also showed that the rate of health anxiety in females (17.76 ± 8.84) was significantly higher than males 15. 96 ± 8. 75 (*P* = 0.03). There was no significant correlation between health anxiety total score and other variables such as marital status, having children, having underlying disease, and career type.

The effects of demographic data on the risk of health anxiety were analyzed using binary logistic regression models. Age (OR 0.682, CI 0.552- 0.843, *P* < 0.001) and hospital category (OR 1.356, CI 1.068–1.722, *P* = 0.013) were significant risk factors. Based on regression results, older HCWs experience less health anxiety compared to younger ones. Also, employees of private and semi-private centers experience more health anxiety than employees of public hospitals. The precision, sensitivity, and specificity of the logistic regression model were 60.32%, 84.56%, and 31.28%, respectively.

## Discussion

In the present study, we have assessed the level of COVID-19-related health anxiety and its predictors in HCWs in hospitals involved in COVID-19. The results showed that the mean score of health anxiety of these employees was higher than the cutoff point of health anxiety, which indicates that participants have a high level of health anxiety. Consistent with this finding, in other studies, the health anxiety of HCWs during the COVID-19 epidemic, was higher than the cutoff point and HCWs had high level of health anxiety [21, 30, 31]. One of the reasons for the high level of health anxiety in HCWs was that this study was conducted in the early months of the COVID-19 outbreak in Iran. The official announcement of the COVID-19 outbreak in Iran was February 19, 2020, and this study was performed about 4 months later. Therefore, this study was performed at the beginning of the outbreak of COVID-19, at a time when many aspects of the disease were still unknown and the vaccine was not widely available.

Another important factor leading to health anxiety in HCWs is the high rate of infection and mortality among them [41]. Medical staff caring for COVID-19 patients; experience mental stress, physical fatigue, separation from family, stigma, and the stress of losing patients and colleagues. Many of them become infected with COVID-19 and some of them die [42]. If these stressors are not effectively addressed, they may not only weaken the staff's immune system and increase the risk of COVID-19 infection, but may also adversely affect the quality and safety of medical services [43]. Studies have reported a high prevalence of COVID-19-related health anxiety in HCWs caring for patients with COVID-19 [5, 19, 44]. A recent Chinese study found that one-sixth of health personnel suffered from mental health problems during the outbreak of COVID-19, of which only 35% sought treatment [5].

In the present study, 59.6% of HCWs had COVID-19-related health anxiety. Consistent with this finding, Ardakani et al. reported that 58.6% of HCWs had COVID-19-related health anxiety [21]. Research shows that health anxiety is higher in HCWs than in the general population [18, 19]. Research on the psychological effects of the spread of infectious diseases such as Acute Respiratory Syndrome (SARS) and pandemic influenza (H1N1) on HCWs shows they are exposed to the stress of overwork due to the outbreak of infectious diseases, fear of transmitting the disease to themselves and their families, working with new and changing protocols, caring for critically ill and dying patients, and caring for their sick colleagues [45–47]. Given that the epidemic of infectious diseases such as COVID-19 may last for months and years, HCWs need to have a long-term health strategy. Therefore, immediate interventions are necessary to strengthen mental resilience and increase the capacity of health care systems [43].

According to the results of the present study, among the demographic variables, age and hospital category are predictors of health anxiety. Based on the results of the present study, there is a significant inverse relationship between the total score of health anxiety and age, so that with increasing age, the amount of health anxiety decreases. Consistent with this finding, in some studies [29, 32, 48, 49], young HCWs had higher health anxiety than older HCWs. However, some studies have not reported a significant relationship between age and health anxiety [20, 21, 33]. Young healthcare workers are experiencing this pandemic for the first time, and their higher anxiety in this study may be due to their inexperience [50].

According to the regression results, employees of private and semi-private centers experience more health anxiety than employees of public hospitals. There was no available study to compare health anxiety among public and private HCWs, but the studies comparing the stress and anxiety of HCWs in public and private hospitals had various results. In the study by Hafiz et al., Private hospital physicians experienced more stress related to patient care responsibilities and professional uncertainty than public sector physicians [51]. In the study by Tyler et al., The stress level of private sector nurses was higher than the public sector ones, but it was not statistically significant [52]. In contrast, in the study of Tyson and Pongruengphant, public sector nurses were more stressed, but their job satisfaction was higher due to improvements in monetary compensation and organizational support [53]. Although most of the COVID-19 management in Iran is on public hospitals, it should be noted that access to protective equipment in public centers is more than in other centers. Inadequate protective equipment is associated with higher levels of stress and anxiety in HCWs [54, 55]. On the other hand, the sensitivity of public hospital staff may have decreased due to high exposure to patients. However, it should be noted that in this study, the number of participants from private and semi-private centers is lower than in public centers and the possibility of selection bias can be raised.

In the present study, the severity of health anxiety was significantly higher in female employees than in men. Consistent with this finding, other studies also reported that the female gender was found as a risk factor for health anxiety [19, 32, 33]. According to the literature, women are more likely than men to develop anxiety disorders [56]. More anxiety in women can be caused by biological factors such as sex hormones and psychosocial factors such as performing more tasks and responsibilities in life. In addition, neurological factors, such as hyperactive extra-hypothalamic corticotropin-releasing factor circuits in women, may explain this gender difference in health anxiety.

Based on the results of this study, work experience and employees' income level had a significant inverse relationship with the total score of health anxiety. Similarly, in the study of Mirzabeigi et al. more experienced HCWs had less health anxiety [20]. More experienced employees have faced similar epidemics such as SARS, MERS, and Ebola, they have more knowledge, and skills, compared to employees with less work experience and are more able to self-regulate, thus they are better able to adapt. Other studies have reported a significant inverse relationship between income levels and anxiety [19]. The Low—income staff is more vulnerable to disease. In addition to illness, staff concerns about treatment costs, job losses, and declining incomes during illness can increase their anxiety.

This study had some limitations. First, due to the prevalence of coronavirus and the expansion of health centers, data collection was done online, which may result in less participation of samples in this method compared to the face-to-face method; Furthermore, the response rate was low, so this may affect the results. Second, the lack of Internet access for some employees to participate in this study was another limitation. Third, inequality in the number of participants in terms of gender, type of job, type of hospital, etc. is another limitation that may affect the generalization of the results. Fourth, various factors can affect health anxiety, in this study, we examined only some of the demographic variables. Further health-related features are needed to fully understand health anxiety in the medical staff.

## Conclusion

Based on the results of this study, employees working in health centers in Iran had high health anxiety. Due to the high level of health anxiety in HCWs, it is important to consider strategies to reduce their health anxiety in the current situation.

## Data Availability

The datasets used or analyzed during the present study are available from the corresponding author on reasonable request.

## Abbreviations

COVID-19: Coronavirus disease 2019; HCWs: health care workers
